# Olfactory Dysfunction as a Biomarker for Early Diagnosis of Cognitive Impairment in Patients With Type 2 Diabetes: A Systematic Review

**DOI:** 10.1155/jdr/9933957

**Published:** 2024-12-19

**Authors:** Paula Ramos-Cazorla, Lina Carazo-Barrios, Jose A. Reyes-Bueno, Elena Sagües-Sesé, Carmen de Rojas-Leal, Miguel A. Barbancho, Francisco J. Garzón-Maldonado, C. de la Cruz-Cosme, Juan A. García-Arnés, Natalia García-Casares

**Affiliations:** ^1^Department of Medicine, Faculty of Medicine, University of Málaga, Málaga, Spain; ^2^Department of Neurology, University Hospital of Jaén, Jaén, Spain; ^3^Department of Neurology, Regional University Hospital of Málaga, Málaga, Spain; ^4^Department of Neurology, University Hospital Virgen de la Victoria of Málaga, Málaga, Spain; ^5^Biomedical Research Institute of Málaga-Nanomedicine Platform (IBIMA-Plataforma BIONAND), Málaga, Spain; ^6^Clinical Neurology Unit, Centro de Investigaciones Médico-Sanitarias (CIMES), Málaga, Spain; ^7^Department of Physiology, Faculty of Medicine, University of Malaga, Málaga, Spain; ^8^Department of Pharmacology and Therapeutics, Faculty of Medicine, University of Malaga, Málaga, Spain

**Keywords:** anosmia, diabetes, mild cognitive impairment, olfactory dysfunction, Type 2 diabetes

## Abstract

**Background:** Olfactory dysfunction and cognitive impairment (CI) have been associated with Type 2 diabetes (T2DM), but the mechanisms underlying this association are broadly unknown. This systematic review tends to investigate the relationship between the onset of olfactory dysfunction and CI in patients with T2DM and to explore the potential role of olfactory dysfunction as an early diagnosis biomarker of CI.

**Methods:** We conducted a systematic review consulting PubMed and Scopus. The articles considered eligible included patients with T2DM and cognitive and olfactory test.

**Results:** The search identified a total of 145 articles, of which 13 were finally selected. The majority of these studies discovered a correlation between olfactory dysfunction and CI in individuals with T2DM. Additionally, other biomarkers such as functional magnetic resonance imaging demonstrated changes in brain regions associated with the sense of smell in T2DM patients.

**Conclusions:** Olfactory dysfunction could be a biomarker for early diagnosis of CI in T2DM. However, these alterations are highly heterogeneous and more studies that include neuroimaging need to be conducted.

## 1. Introduction

Type 2 diabetes mellitus (T2DM) is a public healthcare problem and one of the most frequent diseases worldwide, especially in older patients. The prevalence of the disease is estimated to grow to 4.4% in 2030, compared to 2.8% in 2000 [1]. Expanding the knowledge about T2DM comorbidities and management has become more necessary in this scenario of growing prevalence. One important comorbidity in patients with T2DM is cognitive impairment, which can be present in 45% of patients [2]; recent research has shown that patients with T2DM have a two to four times greater risk of developing cognitive impairment compared to patients with a healthy metabolic situation [3]. A rapid deterioration of cognitive status could negatively impact the disease prognosis, causing difficulties in medication management, worse therapeutic adherence, and poorer compliance with dietary measures. Currently, there are no available treatments capable of slowing or preventing the progression to dementia; therefore, the development of biomarkers for early diagnosis of cognitive impairment is needed in order to provide prompt diagnosis and improve the prognosis.

T2DM carries many microvascular complications, like neuropathy, nephropathy, and retinopathy; these complications can cause severe and irreversible organ damage; therefore, they are subjected to a regular screening. Other symptoms such as olfactory dysfunction are not so well characterized but could also be relevant: Recent research shows that olfactory dysfunction presents 1.58 times more frequently in patients with T2DM [4].

On the other hand, olfactory dysfunction is well known to be one of the earliest manifestations of Alzheimer's disease (AD) and mild cognitive impairment (MCI) [5–10]. Also, the presence of impaired olfactory discrimination can predict the progression of MCI to dementia [11, 12]. Eighty-five percent to 90% of patients with AD show olfactory dysfunction, even in early stages of the disease [13]; the presence of olfactory dysfunction can discriminate between AD and cognitively healthy controls with a sensitivity and specificity of 85% [14].

The precise mechanisms underlying olfactory dysfunction in patients with AD are broadly unknown. Recent research shows that olfactory dysfunction is associated with increasing amyloid-*β* and tau deposit in the olfactory bulb as Braak stages increase [15] and is also associated with a deposit in other brain areas involved in olfaction, such as the piriform cortex and the anterior olfactory nucleus [16–18]. In healthy brains, the olfactory system neurotransmission implies acetylcholine, glutamate, and *γ*-aminobutyric acid (GABA). A decrease in any of these neurotransmitters, especially acetylcholine (cholinergic dysfunction is well documented in AD) [19], could play a role in olfactory dysfunction [20].

Patients with AD may experience accelerated olfactory nerve damage when accompanied by T2DM microvascular damage, compared to nondiabetic AD patients. Additionally, given that AD can present with olfactory dysfunction, the hypothesis that patients with T2DM and olfactory dysfunction could carry a higher risk of cognitive impairment is to be considered. The relationship between other comorbidities of T2DM (neuropathy, nephropathy, or retinopathy) and olfactory dysfunction has also been studied [21–24]. However, although many studies show a clear association between olfactory dysfunction and cognitive impairment, whether the former could serve as a potential biomarker for the latter is still unknown [25].

Developing olfactory dysfunction as a reliable and accessible early diagnosis biomarker for cognitive impairment in patients with T2DM could have important benefits. Olfactory tests are considered optimal for dementia screening because they are simple, effective, and non–time-consuming, typically taking less than 5 min, making them well suited for clinical environments [25–27]. Based on this, the current systematic review seeks to examine the connection between olfaction and cognitive decline in individuals with T2DM. This endeavor is aimed at uncovering the potential utility of olfactory dysfunction as an early diagnostic marker for cognitive impairment within this specific patient cohort.

## 2. Materials and Methods

In order to conduct this systematic review, we followed Preferred Reporting Items for Systematic Reviews and Meta-Analyses (PRISMA) methodology [28].

### 2.1. Search Strategy

PubMed and Scopus databases were consulted. The keywords used were “diabetes,” “Alzheimer,” “cognitive impairment,” “cognition,” “anosmia,” “olfactory dysfunction,” “olfactory impairment,” and “olfaction” and the Boolean operators “AND/OR.” No date restrictions were considered (supporting information (available here)).

After excluding duplicate articles and screening by title and abstract, an initial selection was made. The final selection comprised articles which made reference to olfactory dysfunction, cognitive impairment, and diabetes.

### 2.2. Quality Evaluation

The quality of each observational study was evaluated using the Strengthening the Reporting of Observational Studies in Epidemiology (STROBE) recommendations. These recommendations propose a 22-item verification list to evaluate the data contained in the article [29]. Each verified item accounts for 1 point, and a final score over a maximum of 22 points was calculated for each article and presented in Table 1.

### 2.3. Eligibility and Selection Criteria

In order to be included, the articles needed to fulfill all of the following requirements: (1) at least one group of patients with T2DM, (2) cognitive exploration with at least one validated cognitive test, and (3) at least one validated olfactory test was conducted. Both prospective and cross-sectional studies were included.

We excluded articles that (1) did not fulfill the aims of our study, (2) did not evaluate cognitive and olfactory function simultaneously, and (3) were conducted in animals.

### 2.4. Data Recollection and Analysis

**Figure 1:**
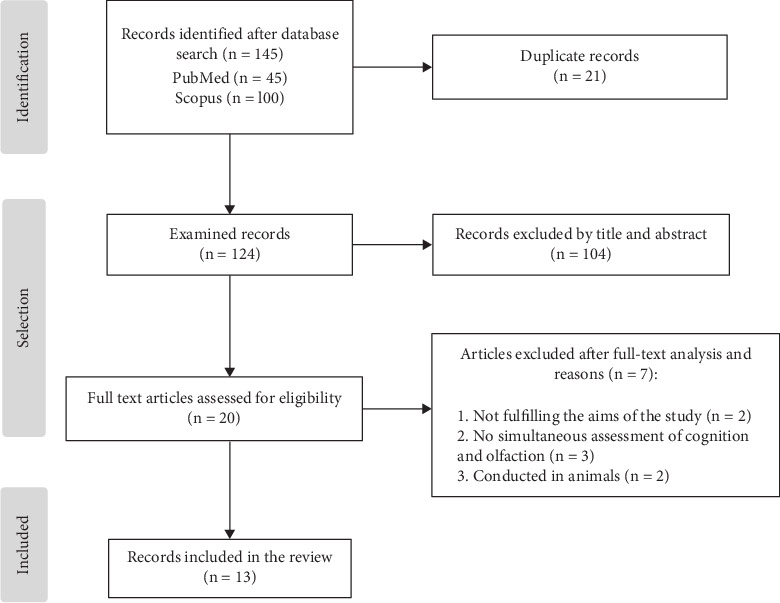
Flowchart of the search strategy.

The selection process is presented in Figure 1. We identified 145 articles in the database search. After eliminating duplicate records, 124 articles were examined, finally selecting 13 for the systematic review. The selection was made after title and abstract review, and a detailed reading of the complete text was performed when required.

## 3. Results

### 3.1. Study Designs

Results of the systematic review are summarized in Table 1 and presented in a chronological order. We present the main author of the study, publication year, type of study, cognitive and neuropsychological tests, olfactory tests, imaging tests when conducted, results, and conclusions of each study. Publications dates range from 2014 to 2023. The age range of the participants is between 47 and 72 years old. Most of the studies originate from China and Japan. Six articles analyzed patients with T2DM alone and did not compare with a control group [25, 30–33], and eight articles compared patients with T2DM with a control group [22, 34–40]; among these eight, two articles performed a subgroup analysis in patients with obesity [34] and peripheral neuropathy (PN) [22]. Eleven studies had a cross-sectional design [22, 30–35, 37–40], and two had a longitudinal and prospective design [25, 36].

### 3.2. Cognition

All of the studies performed at least one cognitive screening test; six used Mini-Mental State Examination (MMSE) [25, 30–32, 36, 40], two used Montreal Cognitive Assessment (MoCA) [33, 37], and five used both [22, 34, 35, 38, 39]. In addition to this, six studies included neuropsychological tests related to anxiety and/or depression [25, 30, 34, 35, 37, 39]. Six studies found cognitive differences between patients with T2DM and healthy controls through the various cognitive and neuropsychological measures; patients in the control group scored higher in cognitive tests [22, 36–40]. On the other hand, two studies did not find these differences [34, 35]. In one of these last two studies, a subgroup analysis was performed in patients with T2DM with and without obesity, showing that obese patients with T2DM showed poorer performances in MMSE test than nonobese patients with T2DM [34].

### 3.3. Olfaction

All of the studies included an olfactory test. Three included Open-Essence (OE) test [25, 30, 37], three included the Connecticut Chemosensory Clinical Research Center (CCCRC) test [31, 32, 40], three included OLFACT test [22, 34, 35], two included the Chinese Smell Identification Test (CSIT) [38, 39], and one included alcohol sniff test [33].

These different tests have distinct methodologies and scoring systems. The OE test uses 12 cards with different smells, requiring participants to identify each odor from two options; a perfect score of 12 indicates accurate identification, while lower scores suggest olfactory dysfunction [25, 27]. The CCCRC test involves presenting eight butanol dilutions, where the final score reflects the highest correctly identified concentration and indicates the degree of olfactory impairment [31]. Additionally, the OLFACT test includes a threshold component with binary dilutions of n-butanol, scoring from 1 to 13.5, and identification/memory tasks where participants identify and recall 20 odors, with a 10-min break between tasks [34].

All of the studies (eight) that included a control group in their design concluded that patients with T2DM performed significantly poorer in olfactory tests than healthy controls [22, 34–40].

Four studies compared patients with T2DM with and without cognitive impairment, and all of them described significantly reduced scores in olfactory tests performed by patients with T2DM and cognitive impairment when compared to patients with T2DM without cognitive impairment [25, 30, 31, 33]. One study also found that this score was significantly lower in patients with dementia compared to patients with MCI [30]. The same author described in a longitudinal trial published afterwards that patients with lower OE scores at the beginning of the study had a higher probability of progressing to dementia after 3 years of follow-up than those who showed normal scores at baseline [25].

Eight studies found a positive correlation between olfactory scores and MMSE and other cognitive test scores in patients with T2DM [25, 30–32, 34, 35, 38, 39]. One of these studies described this positive correlation between olfaction and MMSE score when comparing obese patients with T2DM and nonobese patients with T2DM [34], and another study described the same correlation when comparing patients with T2DM and PN and patients with T2DM without PN [22]. One study determined that this positive correlation could also be applicable to the control group [40], whereas another study defended that the results obtained in patients with T2DM were not applicable to the control group [35].

The short blessed test (SBT) was used as a cognitive measure in a study, and it showed lower scores in patients with lower scores in olfactory tests [36]. Another study found a negative correlation between SBT scores with trail making test A (TMT-A) scores in all patients, while only a positive correlation with MoCA and a negative correlation with trail making test B (TMT-B) scores in patients with T2DM [37].

### 3.4. Neuroimaging

Six studies included neuroimaging tests [22, 34–36, 38–40], and seven did not [25, 30–33, 37, 40]. Among the studies that performed neuroimaging tests, four performed functional magnetic resonance imaging (fMRI) applying a resting-state and odor-induced activation paradigm and measuring activation and functional connectivity and structural MRI [22, 33, 34, 39]. Two studies only performed structural MRI [36, 38]. Among these, five performed 3-T MRI [22, 34, 35, 38, 39] and one performed 1.5-T MRI [36].

All of the studies [22, 34, 35, 39] that performed resting-state and odor-induced fMRI with functional connectivity analysis found statistically significant differences between the study groups, although these differences were highly heterogeneous. Zhang et al. reported a decreased activation of the left hippocampus and left parahippocampus in patients with T2DM compared to the control group, as well as a significantly decreased functional connectivity with the right middle and inferior orbitofrontal cortex in patients with T2DM compared with the control group [35]. A latter study by the same authors showed that patients with T2DM presented a decreased activation of the left hippocampus compared to the control group, being this disruption more significant in obese patients with T2DM than in nonobese patients with T2DM; this study as well concluded that obese patients with T2DM showed disrupted functional connectivity between the left hippocampus and right insula, when compared to nonobese patients with T2DM and the control group [34]. Ni et al. found that patients with T2DM and PN showed decreased activation of the left frontal lobe and decreased functional connectivity with the right insula when compared to T2DM patients without PN and the control group [22]. Lastly, Luo et al. performed MRI with arterial spin labeling (ASL) analysis and observed a significant decrease of cerebral blood flow (CBF) to the right orbital part of the inferior frontal gyrus, right insula, and bilateral olfactory cortex in patients with T2DM compared to the control group, as well as CBF connectivity alterations in patients with T2DM: CBF connectivity between the right orbital part of the inferior frontal gyrus and the left temporal pole of the middle temporal gyrus was decreased, and CBF connectivity was increased between the right medial orbital part of the superior frontal gyrus and the right orbital part of the superior frontal gyrus, as well as between the right olfactory cortex and the bilateral caudate and the left putamen [39].

The two studies that only performed structural MRI did not find significant differences in the cortical thickness of olfactory-related areas, regardless of the imaging quality (1.5 or 3 T) [36, 38]. One of the studies observed no differences in transverse T2 relaxation time between patients with T2DM, pre-T2DM, and controls [36].

Two studies analyzed the relationship between neuroimaging tests, cognitive tests, and olfactory tests and several clinical variables [19, 38]. The first study concluded that higher CSIT scores showed a positive correlation with cortical thickness in the left parahippocampal gyrus, left insula, and right insula in patients with T2DM; left parahippocampal gyrus thickness correlated to higher scores in auditory verbal learning test (AVLT), MoCA, symbol digit modalities test (SMDT), digit span test-backward (DST-backward), and verbal fluency test (VFT); and left insular thickness correlated to MoCA and DST-backward test scores [38]. The second study found that duration of T2DM negatively correlated with blood flow changes in the right orbital part of the inferior frontal gyrus, right insula, and right olfactory cortex; changes in CBF connectivity to the right orbital part of the inferior frontal gyrus showed a positive correlation with scores in Self-Rating Depression Scale (SDS), changes in the right insula showed a negative correlation with maximum blood glucose, and changes in the right olfactory cortex showed a negative correlation with average blood glucose [39].

### 3.5. Other Measurements

Two studies tested a combination of biomarkers for early detection of cognitive impairment, including olfaction test scores, which were useful for improving diagnostic accuracy in patients with T2DM [31, 32]. Both considered the age of patients, ApoE4 *ε*4 gene status, and glycogen synthase kinase-3*β* activity (tGSK-3*β*/pS9GSK-3*β* ratio); Liu et al. also analyzed plasma A*β*1-42/A*β*1-40 levels [32].

There were several studies that included variables related to cognitive and neuropsychological tests. Sanke et al. found a positive correlation between MMSE test and diastolic blood pressure, educational level, OE test score, total cholesterol, LDL cholesterol, and folic acid and a negative correlation with age, HbA1c, aspartate aminotransferase (AST), serum adiponectin, and urinary albumin excretion [30]. Zhang et al. described in another study that higher levels of peptide C correlated with better scores in cognitive tests [35]. Yulug et al. found a correlation between HbA1c levels and the score in letter fluency test (LFT) [36]. Gong et al. described a correlation between scores in cognitive tests and age, educational level, severity, and duration of T2DM [33]. A latter study by Sanke et al. observed, after 3 years of follow-up, that risk factors associated with progression to probable dementia were a low educational level, lower MMSE and OE scores at baseline, higher serum total protein levels, higher leptin, and higher urinary albumin excretion [25]. Midorikawa et al. found a significant correlation between balance capability and all of the cognitive tests they performed except Quick Inventory of Depressive Symptomatology (QIDS) and Self-Rating Anxiety Scale (SAS), both in patients with T2DM and healthy controls, and also a correlation with TMT-A in healthy controls. They also described a correlation between knee extension strength and SAS in the whole cohort [37]. Lastly, Ni et al. studied patients with T2DM and NP and found an association with nerve conduction velocities and cognitive function in patients with T2DM [22].

Three studies found an association between olfactory function and other variables. One found a positive correlation between higher scores in olfaction tests and higher peptide C levels [35]. Another study found an association between random glucose levels and olfactory threshold and an association between HbA1c levels and olfactory discrimination, olfactory identification, and olfactory threshold [36]. The last study described that olfaction influences nerve conduction velocities assessed with electromyography and executive function [22].

## 4. Discussion

The aim of this systematic review was to investigate the relationship between olfactory dysfunction and cognitive impairment in patients with T2DM. The 13 studies we analyzed concluded that olfactory dysfunction was related to a worse cognitive function in T2DM patients [22, 25, 30–40].

Some studies reported that cognitive function declines 50% faster in T2DM patients than it does in normal cognitive aging population [41]. Six of the studies included in this review are aimed in this direction: They reported lower cognitive scores in patients with T2DM than in the control group [22, 36–40]. These cognitive differences were even more pronounced in obese patients with T2DM than in nonobese patients with T2DM [34]. Obesity has also been described as a risk factor to develop AD [42].

All of the studies that described these cognitive differences described as well worse olfactory performances [22, 36–40], which suggests that cognitive impairment was previously present or that it was not preceded by olfactory dysfunction. Nevertheless, there is conflicting evidence at this point: None of the studies by Zhang et al. described measurable differences in cognitive scores comparing patients with T2DM and controls, but they did find differences in olfactory scores [34, 35]. Moreover, cross-sectional studies are limited by their inability to establish causal relationships.

Considering both approaches, cognitive and olfactory scores, most of the studies found a positive correlation between olfactory dysfunction and cognitive scores in patients with T2DM. The main cognitive test used was MMSE [25, 30–32, 34, 35, 38, 39]. Gao et al. maintain that these results would be applicable to patients with T2DM and to community-dwelling elderly as the control group as well [40], which showed higher cognitive scores at baseline. This difference could be of interest for studying the association between olfactory dysfunction and cognitive impairment in the general population [7, 9, 10].

The underlying mechanism that links olfactory dysfunction and cognitive impairment in patients with T2DM is unclear [25]. Insulin receptors are widely expressed across the whole brain, especially in brain areas related to olfaction: olfactory epithelium, olfactory bulb, and hippocampus [43, 44]. Insulin levels in brain tissue could yield an important role both for olfactory and for cognitive functions [45]. In fact, there are several studies that report an improvement of cognitive function after administering intranasal insulin in healthy patients [46] and in patients with T2DM [47] and an association between olfactory dysfunction and insulin resistance [48, 49].

Nonetheless, determining whether olfactory dysfunction appears before or after cognitive impairment remains an unsolved question. Our systematic review includes 11 cross-sectional studies [22, 30–35, 37–40] and only two longitudinal and prospective studies [25, 36]. This calls for caution when drawing conclusions and makes this question harder to solve. Although the study by Sanke et al. described that olfactory dysfunction precedes cognitive impairment, extrapolating the same probable progression is not possible: Olfactory dysfunction could not be a cause, but a consequence of cognitive deterioration. Some authors also propose that olfactory dysfunction could be another clinical expression of diabetic neuropathy [49].

In any case, olfactory evaluation using validated tests like OE [27], CCCRC [50], or CSIT [51] could be a useful tool for early detection of cognitive impairment in patients with T2DM. Although these tests are widely available and have a low cost, thus being optimal for this evaluation, they are subjective measurements requiring patient collaboration.

On the other hand, neuroimaging tests such as MRI and fMRI are noninvasive tests able to describe cerebral structures related to olfaction, their structure, and functioning. Several alterations have been described in patients with T2DM via the use of diffusion tensor imaging (DTI); these alterations have already been described in this review, and they include fractional anisotropy reduction in the left olfactory cortex, among others [52]. Six of the studies included in this systematic review included neuroimaging tests [22, 34–36, 38–40], but only the four studies that performed fMRI with activation and functional connectivity analysis found significant differences in olfaction between patients with T2DM and controls [22, 33, 34, 39]. These differences were very heterogeneous; therefore, more studies are needed to concrete and precisely define these alterations via neuroimaging tests. These limitations add to the limited accessibility of fMRI, hampering the use of fMRI as a tool for early detection of cognitive impairment, at least for the moment.

The results of this systematic review suggest that there is a relationship between olfactory dysfunction and cognitive impairment in patients with T2DM. Validated olfactory tests to detect olfactory dysfunction are a simple, affordable, and efficient tool that could serve as biomarkers for early detection of cognitive impairment or even predicting progression to dementia, allowing early treatments. Similarly, neuroimaging tests for early detection of cognitive impairment could be an interesting tool in the future, although they present limited availability and accessibility. Neuroimaging could constitute a valuable tool for describing structural alterations in brain areas related to olfaction and cognitive impairment. Nonetheless, whether olfactory dysfunction is a cause or a consequence of this cognitive impairment is yet to be determined. More studies with a longitudinal and prospective design are needed to study the temporal progression of both clinical events: If olfactory dysfunction starts before cognitive impairment, we could be facing a new therapeutic target to which investigatory efforts could be directed, given the absence of available pharmacotherapy to slow progression to dementia.

## Figures and Tables

**Table 1:** Characteristics and results of the studies.

**Author/study, year, country**	**Participants**	**Research design**	**Mean age**	**Cognitive and neuropsychological tests**	**Olfactory tests**	**Neuroimaging tests**	**Results**	**Conclusions**	**STROBE score**
				**Other measures**					
Sanke et al. [30] 2014 Japan	*n* = 250 T2DM No relevant CI	Cross-sectional	72	MMSE BDI-II	OE		62% of participants showed no signs of cognitive impairment, 24.4% had possible CI, and 13.6% had probable dementia, according to MMSE score Educational level was significantly lower in groups with probable CI and probable dementia, compared to participants without CI (*p* < 0.001) OE score was significantly lower in participants with probable dementia compared to participants with possible CI and no CI, and OE score was significantly lower in participants with CI compared to participants without CI (*p* < 0.001) MMSE score showed a positive correlation with diastolic BP, educational level, OE score, total and LDL cholesterol, and folic acid. MMSE score showed a negative correlation with age, HbA1c, AST, seric adiponectin and UAE OE score showed a significant and independent correlation with MMSE score (*p* < 0.01)	Association between olfactory dysfunction and cognitive dysfunction severity in T2DM patients	20
Xu et al. [31] 2016 China	*n* = 646 Training set (*n* = 345, 85 T2DM with MCI vs. 260 T2DM without MCI) Validation set (*n* = 301, 100 T2DM with MCI vs. 201 T2DM without MCI)	Cross-sectional	65	MMSE CDR Petersen criteria for MCI diagnosis ApoE *ε*4 genotyping	CCCRC		T2DM with MCI showed significantly lower scores in MMSE, higher ApoE *ε*4 prevalence, higher rGSK-3*β*, and higher olfactory scores when compared to T2DM without MCI in the training set and in the validation set (*p* < 0.05) Strong and significant negative correlation between olfactory scores and MMSE scores in T2DM patients (*p* < 0.001) The diagnostic accuracy of age, ApoE *ε*4 allele status, olfactory scores, and rGSK-3*β* was 1.09, 2.09, 1.51, and 10.08 in the training set and 1.06, 2.67, 1.47, and 7.19 in the validation set, respectively These four combined biomarkers showed an AUC of 82% and 86% and diagnostic accuracy of 83% and 81% in the training set and the validation set, respectively	A higher olfactory score can serve as a diagnostic tool for MCI detection in T2DM patients, as well as age and ApoE4 *ε*4 activation of peripheral circulating GSK-3*β* The combination of these biomarkers can be useful for early MCI detection in T2DM patients	20
Zhang et al. [35] 2018 China	*n* = 92 (52 T2DM vs. 41 healthy controls)	Cross-sectional	51	MMSE, MoCA, 12-word Chinese version of the PVLT, WMS, TMT-A–B, DST, BNT, ANT, SCWT (I, II, and III) HDRS, HIS, CDR®	Olfactory threshold, identification, and memory test	MRI 3 T and fMRI: resting-state and odor induced (activation and functional connectivity analysis)	No differences in measured cognitive domains when comparing both groups (*p* < 0.05) T2DM patients showed lower olfactory threshold test scores, although they remained in the range of normality (*p* = 0.010) Odor-induced fMRI revealed lower hippocampal and parahippocampal activation (left side) comparing patients with T2DM and controls (*p* < 0.05) Functional connectivity of the right inferior and middle orbitofrontal cortex was significantly lower in T2DM patients compared to the control group (*p* < 0.05) Olfactory test scores and cognitive scores showed a positive association in patients with T2DM, not in the control group (*p* < 0.0071) Higher levels of peptide C showed a positive correlation with higher cognitive and olfactory scores (*p* < 0.05)	Lower scores in olfactory tests and alterations in brain olfactory circuit start before changes in brain structure and clinically measurable cognitive impairment in T2DM patients with normal cognitive status	21
Zhang et al. [34] 2019 China	*n* = 105 (70 T2DM (35 obese and 35 nonobese) vs. 35 controls)	Cross-sectional	51	MMSE, MoCA, 12-word Chinese version of the PVLT, WMS, TMT-A–B, DST, BNT, ANT, SCWT (I, II, and III) HDRS, HIS, CDR®	Olfactory threshold, identification, and memory test (OLFACT™)	MRI 3 T and resting-state and odor-induced fMRI (activation and functional connectivity analysis)	Obese patients with T2DM showed significantly lower MMSE scores compared to nonobese patients with T2DM (*p* = 0.014) Patients with T2DM and normal cognitive function scored lower in the olfactory tests compared to the control group (*p* = 0.035) Obese patients with T2DM scored significantly lower in olfactory tests compared to nonobese patients with T2DM (*p* = 0.028) fMRI showed decreased activation of the left hippocampus in patients with T2DM compared to the control group, more important in obese T2DM patients than in nonobese T2DM patients (*p* < 0.05) Functional connectivity was significantly decreased between the left hippocampus and right insula in obese T2DM patients compared to nonobese T2DM patients and the control group (*p* < 0.05)	Obese patients with T2DM present with a more important cognitive and olfactory impairment, as well as disruptions in odor-induced brain activity when compared to nonobese T2DM patients Preclinical olfactory and cognitive dysfunction improves with aGLP1 treatment in obese T2DM patients Olfactory dysfunction can be a good early biomarker of cognitive dysfunction in patients with T2DM	21
Yulug et al. [36] 2020 Turkey	*n* = 46 (15 healthy vs. 16 pre-T2DM vs. 15 T2DM)	Longitudinal	51	MMSE, SBT, LFT, COWAT-KAS, CFT	Sniffin' Sticks test battery (Burghart Messtechnik, Wedel, Germany)	MRI 1.5 T	Significant decrease of OD, OI, OT, and cognitive scores when comparing patients with T2DM and pre-T2DM to healthy controls (*p* < 0.05) SBT was the only test significantly correlated with altered OI in pre-T2DM and T2DM groups (*p* < 0.05) Random glucose values correlated with OT (*p* < 0.05) HbA1c levels correlated with OT, OI, OD, and LFT scores (*p* < 0.05) No signs of hippocampal atrophy or hippocampal sclerosis among the participants. No differences in hippocampal volumes or transverse T2 relaxation time between the three groups	Strong association between olfactory dysfunction and cognitive impairment in pre-T2DM and T2DM patients	20
Sanke et al. [25] 2021 Japan	*n* = 151 T2DM (112 no CI, 39 possible CI)	Longitudinal	71	MMSE BDI-II	OE		After 3 years of follow-up, 9% of participants progressed to probable dementia Participants with olfactory dysfunction at baseline were at a higher risk of developing dementia than those with a normal olfactory function at baseline MLR showed that lower OE scores, lower MMSE scores, age, total seric protein concentration, and more frequent use of sulphonylureas presented a significant association with the development of probable dementia Baseline MMSE score and OE score changes during the 3 years of follow-up showed a significant association with MMSE score changes (*p* < 0.01)	Olfactory dysfunction precedes progression to dementia in patients with T2DM and no diagnosis of dementia at baseline	20
Ni et al. [22] 2021 China	*n* = 100 (36 healthy controls vs. 36 T2DM without PN vs. 28 T2DM with PN)	Cross-sectional	53	MMSE, MoCA, PVL, WMS, TMT-A–B, ANT, BNT, SCWT (I, II, and III), DST EMG	Olfactory Function Assessment by Computerized Testing (OLFACT™)	MRI 3 T and resting-state and odor-induced fMRI (activation and functional connectivity analysis)	T2DM with the PN group showed significantly lower scores in memory tests (*p* = 0.042) and processing speed tests (TMT-A and TMT-B) (*p* = 0.004), when compared to participants with T2DM without PN T2DM with PN participants showed significantly lower scores in olfactory identification and olfactory memory tests (*p* < 0.05) T2DM with PN showed significantly lower olfactory identification and olfactory memory scores (*p* < 0.05) T2DM with PN showed decreased activation of the left frontal lobe and decreased functional connectivity to the right insula (*p* < 0.05) Nerve conduction velocity in participants with T2DM showed an association with cognitive function. The association with executive function was mediated by olfaction	T2DM patients showed lower scores in cognitive and olfactory tests, as well as decreased activation of brain areas related to olfaction, when compared to the control group. These differences were more important in patients with T2DM and PN	20
Gong et al. [33] 2021 China	*n* = 472 T2DM (162 MCI vs. 310 without MCI)	Cross-sectional	63	MoCA	Alcohol sniff test		Statistically significant reduction of OT in patients with T2DM with MCI compared to patients with T2DM without MCI (*p* < 0.05) 74.1% of patients with T2DM with MCI presented olfactory dysfunction; 16.1% of patients with T2DM without MCI showed olfactory dysfunction Age, educational level, severity of T2DM, duration of T2DM, and the presence of olfactory dysfunction are all independent factors that influence T2DM combined with MCI (*p* < 0.05)	Olfactory dysfunction is an independent risk factor for T2DM with MCI	18
Midorikawa et al. [37] Japan 2021	*n* = 151 (70 T2DM vs. 81 controls)	Cross-sectional	53	MoCA, TMT-A–B, KWCST QIDS, SAS OLST, TUG, IPS, dynamometer, torsion test machine	OE		Participants with T2DM showed significantly lower OE scores and cognitive scores when compared to the control group (*p* < 0.001) OE score showed a significant and independent negative correlation with TMT-A score in all groups. In the T2DM group, OE score was positively correlated with MoCA and negatively correlated with TMT-B scores (*p* < 0.05) Significant correlation of balance capability with all cognitive measures except QSID and SAS, across the whole cohort, and correlation with TMT-A score in controls (*p* < 0.05) Knee extension strength shows independent association with SAS across the whole cohort and in patients with T2DM	Odor identification, balance capability, and knee extension strength were significantly reduced in patients with T2DM Odor identification, balance capability, and knee extension strength were also potential biomarkers of MCI in patients with T2DM	20
Liu et al. [32] 2021 China	*n* = 852 T2DM	Cross-sectional	66	MMSE ELISA (A*β*1-40 and A*β*1-42) ApoE genotyping Dot Blot (GSK-3*β*)	CCCRC		High plasma levels of A*β*1-42/A*β*1-40 are an independent risk factor for MCI in patients with T2DM The combined analyses of A*β*1-42/A*β*1-40 levels, GSK-3*β* levels, ApoE *ε*4 genotyping, olfactory dysfunction, and age show a better discriminative performance between T2DM patients with and without MCI; AUC of 0.846 (95% CI: 0.794–0.897) to 0.869 (95% CI: 0.822–0.916) in the training set and an AUC of 0.848 (95% CI: 0.815–0.882) to 0.867 (95% CI: 0.835–0.899) in the validation set	High A*β*1-42/A*β*1-40 plasma levels, high GSK-3*β* levels, ApoE *ε*4 genotyping, olfactory dysfunction, and age could be efficient diagnostic biomarkers of MCI in patients with T2DM	21
Chen et al. [38] 2022 China	*n* = 136 (68 T2DM vs. 68 controls)	Cross-sectional	47	MMSE, MoCA, AVLT, TMT-A, SDMT, DST, VFT	CSIT-self and CSIT-olfactory impairment	MRI 3 T	T2DM patients showed significantly lower cognitive scores in tests like AVLT (*p* = 0.025), MoCA (*p* = 0.001), SMDT (*p* = 0.001), DST-forward (*p* = 0.018), DST-backward (*t* = 0.029), and VFT (*p* = 0.005) compared to controls T2DM patients showed significantly lower CSIT-OI scores (*p* = 0.020), but no differences were found in the CSIT-self test (*p* = 0.396) No differences in cortical thickness of brain areas related to olfaction between T2DM patients and controls Higher CSIT scores showed a positive correlation with cortical thickness in the left parahippocampal cortex (*p* = 0.030), left insula (*p* = 0.040), and right insula (*p* = 0.030) in patients with T2DM Higher olfactory scores were associated with better cognitive performance in patients with T2DM (*p* < 0.05) Cortical thickness in the left parahippocampal cortex showed a positive correlation with AVLT scores (*p* = 0.010), MoCA (*p* = 0.026), SMDT (*p* = 0.010), DST-backward (*p* = 0.018), and VFT (*p* = 0.039) Cortical thickness in the left insula showed a positive correlation with MoCA (*p* = 0.010) and DST-backward scores (*p* = 0.010)	Olfactory dysfunction could be a useful tool for predicting cognitive impairment or developing personalized therapies in patients with T2DM	20
Luo et al. [39] 2022 China	*n* = 132 (69 T2DM vs. 39 controls)	Cross-sectional	46	MMSE, MoCA, AVLT, TMT-A, VFT, SDMT, DST SDS, SAA	China Smell Identification Test (CSIT)	MRI 3 T (ASL + functional connectivity analysis)	MoCA, DST, SDMT, and VFT scores were significantly lower in patients with T2DM compared to controls (*p* < 0.05) CSIT scores were significantly lower in patients with T2DM compared to the control group (*p* < 0.05) CBF to the OIFG, right insula, and bilateral olfactory cortex was significantly decreased in patients with T2DM compared to controls (*p* < 0.05) T2DM duration showed a negative correlation with CBF changes to the OIFG, right insula, and right olfactory cortex CBF connectivity changes to the OIFG showed a positive correlation with SDS. CBF changes to the right insula showed a negative correlation with maximal blood glucose. CBF changes of the right olfactory cortex showed a negative correlation with average glucose levels Patients with T2DM showed reduced CBF connectivity between the OIFG and the left temporal pole of the middle temporal cortex and increased CBF connectivity between the right medial orbital part of the superior frontal gyrus and right orbital part of the superior frontal gyrus and between the right olfactory cortex and bilateral caudate and left putamen	CBF and CBF connectivity are altered in multiple brain areas related to olfaction in patients with T2DM. These alterations could be a potential neuroimaging biomarker for predicting olfactory dysfunction and cognitive impairment in this group of patients	20
Gao et al. [40] 2023 China	*n* = 2375 (1139 T2DM inpatients [discovery cohort] vs. 1236 community-dwelling elderly [validation cohort])	Cross-sectional	71	MMSE	CCCRC		Regression analysis showed that a reduced OTS was correlated with cognitive impairment (reduced MMSE score) in both cohorts ROC analysis showed that OTS could distinguish CI from normal cognition, with AUC values of 0.71 (0.67, 0.74) and 0.63 (0.60, 0.66), respectively, but was unable to distinguish dementia from MCI Cut-off point number 3 showed the highest validity for screening, showing a diagnostic accuracy of 73.3% and 69.5%	Reduced OTS is associated with CI in T2DM patients and community-dwelling elderly OTS may be used as an accessible screening tool for cognitive impairment screening	—

Abbreviations: ANT: animal naming test; ASL: arterial spin labeling; AST: aspartate aminotransferase; AUC: area under the curve; AVLT: auditory verbal learning test; BDI-II: Beck Depression Inventory II; BNT: Boston Naming Test; CBF: cerebral blood flow; CCCRC: Connecticut Chemosensory Clinical Research Center; CDR: Clinical Dementia Rating; CFT: cognitive function test; CI: cognitive impairment; CI: confidence interval; COWAT-KAS: Controlled Oral Word Association Test; CSIT: Chinese Smell Identification Test; DBP: diastolic blood pressure; DM1: type 1 diabetes mellitus; T2DM: type 2 diabetes mellitus; DST: digit span test; EMG: electromyography; fMRI: functional magnetic resonance imaging; GDS: Global Deterioration Scale; GSK-3*β*: glycogen synthase kinase-3*β*; HDRS: Hamilton Depression Rating Scale; HIS: Hachinski Ischemic Score; IPS: index of postural stability; KWCST: Wisconsin Card Sorting Test (Keio version); LDL: low-density lipoprotein; LFT: letter fluency test; MCI: mild cognitive impairment; MLR: multivariate logistic regression; MMSE: Mini-Mental State Examination; MoCA: Montreal Cognitive Assessment; MRI: magnetic resonance imaging; OD: olfactory discrimination; OE: Open Essence; OI: olfactory identification; OIFG: right orbital part of the inferior frontal gyrus; OLST: one-leg standing time; OT: olfactory threshold; OTS: olfactory threshold score; PN: peripheral neuropathy; PVL: Philadelphia Verbal Learning Test; QIDS: Quick Inventory of Depressive Symptomatology; ROC: receiver operating characteristic; SAA: Self-Rating Anxiety Assessment; SAS: Self-Rating Anxiety Scale; SBT: Short Blessed Test; SCWT: Stroop Color-Word Test; SDMT: Symbol Digit Modalities Test; SDS: Self-Rating Depression Scale; SVF: semantic verbal fluency; TMT-A–B: trail making test A–B; TUG: timed up and go test; UAE: urinary albumin excretion; VFT: verbal fluency test; WMS: Wechsler Memory Scale.

## Data Availability

The authors confirm that the data supporting the findings of this study are available within the articles included in our references.

## Supporting Information

Supporting Information Additional supporting information can be found online in the Supporting Information section. Search strategy in PubMed and Scopus: This supplementary material outlines the search strategy used to explore the relationship between diabetes, cognitive impairment, and olfactory dysfunction. The following search queries were employed in both PubMed and Scopus databases to retrieve relevant studies: (diabetes) AND (cognitive impairment) AND (anosmia), (diabetes) AND (cognition) AND (anosmia), (diabetes) AND (cognitive impairment) AND (olfactory dysfunction), (diabetes) AND (cognition) AND (olfactory dysfunction), (diabetes) AND (cognitive impairment) AND (olfactory impairment), (diabetes) AND (cognition) AND (olfactory impairment), (diabetes) AND (cognitive impairment) AND (olfaction), (diabetes) AND (cognition) AND (olfaction).
