# Supplementary material for: Olfactory Dysfunction as a Biomarker for Early Diagnosis of Cognitive Impairment in Patients With Type 2 Diabetes: A Systematic Review
Source: J Diabetes Res. 2024 Dec 19;2024:9933957. doi: 10.1155/jdr/9933957 (PMC11681984; doi:10.1155/jdr/9933957)
Supplement: Supporting Information — Additional supporting information can be found online in the Supporting Information section. Search strategy in PubMed and Scopus: This supplementary material outlines the search strategy used to explore the relationship between diabetes, cognitive impairment, and olfactory dysfunction. The following search queries were employed in both PubMed and Scopus databases to retrieve relevant studies: (diabetes) AND (cognitive impairment) AND (anosmia), (diabetes) AND (cognition) AND (anosmia), (diabetes) AND (cognitive impairment) AND (olfactory dysfunction), (diabetes) AND (cognition) AND (olfactory dysfunction), (diabetes) AND (cognitive impairment) AND (olfactory impairment), (diabetes) AND (cognition) AND (olfactory impairment), (diabetes) AND (cognitive impairment) AND (olfaction), (diabetes) AND (cognition) AND (olfaction). [file 9933957.f1.docx]

**Olfactory Dysfunction as a Biomarker for Early Diagnosis of Cognitive Impairment in Patients with Type 2 Diabetes: A Systematic Review**

Paula Ramos-Cazorla ^1^, Lina Carazo-Barrios ^2^ , Jose A. Reyes-Bueno ^3^ , Elena Sagües-Sesé^1^, Carmen de Rojas-Leal ^4,5^ , Miguel A. Barbancho ^1,5,6^, Francisco J. Garzón-Maldonado ^4,5^, De la Cruz Cosme C^4,5^, Juan A. García-Arnés ^1^, Natalia García-Casares*^1,5,6^

**SUPPLEMENTARY MATERIAL**

**Search strategy in Pubmed and Scopus**

(diabetes) AND (cognitive impairment) AND (anosmia)

(diabetes) AND (cognition) AND (anosmia)

(diabetes) AND (cognitive impairment) AND (olfactory dysfunction)

(diabetes) AND (cognition) AND (olfactory dysfunction)

(diabetes) AND (cognitive impairment) AND (olfactory impairment)

(diabetes) AND (cognition) AND (olfactory impairment)

(diabetes) AND (cognitive impairment) AND (olfaction)

(diabetes) AND (cognition) AND (olfaction)
